# Time-Restricted Feeding in Mice Prevents the Disruption of the Peripheral Circadian Clocks and Its Metabolic Impact during Chronic Jetlag

**DOI:** 10.3390/nu13113846

**Published:** 2021-10-28

**Authors:** Louis Desmet, Theo Thijs, Rosalie Mas, Kristin Verbeke, Inge Depoortere

**Affiliations:** 1 Translational Research Center for Gastrointestinal Disorders, Gut Peptide Research Lab, University of Leuven, Gasthuisberg, 3000 Leuven, Belgium; Louis.desmet@kuleuven.be (L.D.); Theo.thijs@kuleuven.be (T.T.); r.mas@outlook.be (R.M.); 2 Translational Research Center for Gastrointestinal Disorders, Laboratory of Digestion and Absorption, University of Leuven, Gasthuisberg, 3000 Leuven, Belgium; Kristin.verbeke@kuleuven.be

**Keywords:** chronic jetlag, circadian clock, ghrelin, short-chain fatty acids, time-restricted feeding

## Abstract

We used time-restricted feeding (TRF) to investigate whether microbial metabolites and the hunger hormone ghrelin can become the dominant entraining factor during chronic jetlag to prevent disruption of the master and peripheral clocks, in order to promote health. Therefore, hypothalamic clock gene and *Agrp/Npy* mRNA expression were measured in mice that were either chronically jetlagged and fed ad libitum, jetlagged and fed a TRF diet, or not jetlagged and fed a TRF diet. Fecal short-chain fatty acid (SCFA) concentrations, plasma ghrelin and corticosterone levels, and colonic clock gene mRNA expression were measured. Preventing the disruption of the food intake pattern during chronic jetlag using TRF restored the rhythmicity in hypothalamic clock gene mRNA expression of *Reverbα* but not of *Arntl*. TRF countered the changes in plasma ghrelin levels and in hypothalamic *Npy* mRNA expression induced by chronic jetlag, thereby reestablishing the food intake pattern. Increase in body mass induced by chronic jetlag was prevented. Alterations in diurnal fluctuations in fecal SCFAs during chronic jetlag were prevented thereby re-entraining the rhythmic expression of peripheral clock genes. In conclusion, TRF during chronodisruption re-entrains the rhythms in clock gene expression and signals from the gut that regulate food intake to normalize body homeostasis.

## 1. Introduction

The circadian system rhythmically regulates several gastrointestinal processes enabling organisms to adapt to the daily cycles of nutrient availability [1]. The circadian system involves a central or master clock, located in the suprachiasmatic nucleus (SCN), and peripheral clocks, present in almost every cell of the body. The master clock synchronizes the peripheral clocks to its “standard time”, which is mainly generated by external light cues [2]. Neuronal signals; circulating hormones; and metabolites such as glucocorticoids, for example, corticosterone, ensure communication between the SCN and the peripheral clocks [1,3]. Additionally, the peripheral clocks are also synchronized by more local cues, such as nutrients, that control the rhythms in physiology and behavior [4,5]. Some of these food-dependent zeitgebers can feedback phase information to the master clock. This equilibrium results in balanced metabolic functions involving a coordinated response in multiple organs [1].

In mammals, the core of the molecular clockwork is a transcription–translation feedback loop (TTFL) inducing 24 h rhythms of gene expression. The positive regulators CLOCK and ARNTL form a heterodimer and bind to E-boxes present in genes encoding the negative regulators PER, CRY, and REVERB. These, in turn, repress the activity of the CLOCK-ARNTL heterodimer. In addition, these positive and negative regulators control the expression of the so-called clock-controlled genes (CCGs) responsible for rhythmic output signals by acting on E-boxes and/or ROR response elements present in their regulatory regions [6]. 

A disruption of the circadian system, also called chronodisruption, occurs when exogenous and endogenous effectors disrupt the timing of physiological functions. Common chronodisruptors include light at night and a disturbed eating pattern, as occurs, for example, in shift work, social jetlag, and chronic jetlag [1,7]. When feeding occurs at an unanticipated time, the master clock becomes uncoupled from peripheral clocks to realign with mealtime [8,9,10]. However, the suboptimal expression of circadian-driven genes compromises metabolic homeostasis. Studies in both humans and mouse models of chronodisruption (genetic or environmental) showed an increase in body mass, a loss in rhythmic physical activity, a dampening of the rhythmicity in the respiratory exchange ratio, glucose intolerance, and dyslipidemia [11,12,13,14,15,16]. Further, chronodisruption is also associated with diseases such as stroke, Alzheimer’s disease, and breast and prostate cancer [11,17,18,19,20,21]. 

Time-restricted feeding (TRF) or eating (TRE) (when referring to humans) is an eating pattern where the eating window is restricted to a certain time window. This can be used to restore disruptions in the food intake pattern that will reinforce rhythmic activation of clock genes to promote health [22,23]. A recent randomized controlled trial showed that TRE in obese patients reduced body mass, energy intake, insulin resistance, oxidative stress, and cardiometabolic health [24]. Similar findings were reported in patients with prediabetes [25]. Further, studies using TRF in mice also showed some promising results in the protection against obesity and obesity related conditions such as adiposity, liver steatosis, glucose intolerance, increased serum cholesterol, and reduced bile acid production [26]. 

Animals with time-restricted access to food show strong food-anticipatory activity (FAA) before the expected time of food availability [27]. This FAA is independent of the presence of the SCN, suggesting that it is controlled by a food-entrainable oscillator [28,29]. The hunger hormone ghrelin, produced by the stomach, has been shown to increase FAA and to regulate the timing of food intake by inducing the release of AgRP and NPY in the arcuate nucleus [30,31]. In *Arntl*^−/−^ mice, diurnal rhythms in plasma ghrelin levels and in food intake are abolished, indicating an important interplay between ghrelin, clock genes, and food intake [32,33]. Ablation of AgRP/NPY neurons also impairs adaptations to restricted feeding [34]. Taken together, these findings indicate that ghrelin and AgRP/NPY neurons are important components of the putative food entrainable oscillator during time-restricted feeding. 

Timing of food intake is an important factor in the production of several microbial metabolites. Both in humans and mice, the intestinal microbiota and their metabolites display diurnal oscillations that are under influence of feeding rhythms [35,36,37]. Microbial metabolites such as short-chain fatty acids (SCFA) and bile acids are important entraining factors of peripheral clocks [16,38]. 

We previously have shown that chronic jetlag in mice disturbed the rhythm in feeding patterns, which correlated with changes in the diurnal rhythms of microbial SCFA levels that affected gut clocks and downstream genes [16]. In the present study, we aim to investigate using an intervention with TRF during the induction of chronic jetlag whether food is an entraining factor during chronodisruption that (1) affects alterations in central clock genes; (2) triggers the food entrainable oscillator, ghrelin, in the stomach to stimulate food intake; (3) feeds back to the hypothalamus to prevent alterations in rhythmicity in *Npy*/*Agrp* expression; and (4) prevents the alterations in fecal SCFA levels induced by chronodisruption that, in turn, strengthen/resynchronize the rhythmic activation of the peripheral clock genes to promote health. To assess this, we used three groups of mice: a group of mice that was jetlagged and fed ad libitum (JL AL), a group of mice that was jetlagged and fed a time-restricted feeding diet (JL RF), and a group of control mice fed a time-restricted feeding diet (Ctrl RF).

## 2. Materials and Methods

### 2.1. Mice

Wild-type C57BL/6J mice were obtained at the age of 12 weeks from Janvier Labs (Le Genest Saint Isle, France). Mice had ad libitum access to water and chow (unless states otherwise) and were housed in a temperature-controlled environment. All experiments were approved by the Ethical committee for Animal Experiments of the KU Leuven and carried out in accordance with the approved guidelines (P187/2017).

### 2.2. Experimental Design

#### 2.2.1. Chronic Jetlag Model

**Figure 1:**
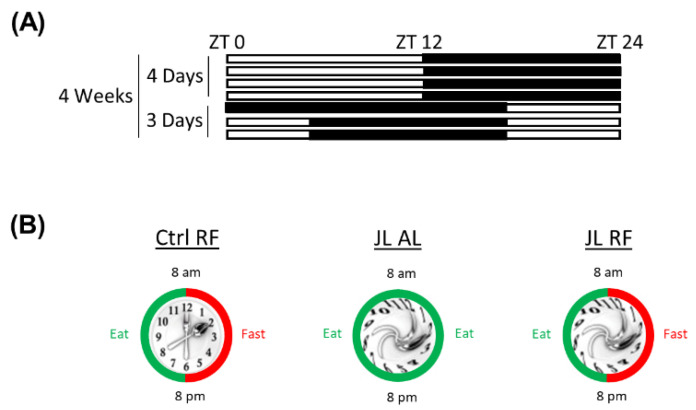
(**A**) Schematic representation of the jetlag model. The light phase is represented in white; the dark phase is represented in black. ZT = Zeitgeber time. (**B**) Graphic representation of control mice fed a TRF diet (Ctrl RF), jetlagged mice fed ad libitum (JL AL), and jetlagged mice fed a TRF diet (JL RF).

Control mice were kept under a 12 h light/12 h dark cycle (12:12 LD) (ZT 0 = lights on). Jetlagged mice were housed for four days a week under a 12:12 LD cycle (ZT 0 = lights on) and the remaining three days of the week under an eight hour advanced 12:12 LD-cycle (ZT 8 = lights on). Chronic jetlag induction lasted for four consecutive weeks (Figure 1A). Zeitgeber time (ZT) refers to the time at which an environmental cue entrains the circadian clock, such as light/dark or feeding/fasting.

#### 2.2.2. Time-Restricted Feeding Model

Mice were randomly assigned to three groups each including 48 mice (N = 8 mice per time point per group) (Figure 1B). The groups were a jetlag group that was fed a time-restricted feeding diet (JL RF), a second jetlag group that had ad libitum access to food (JL AL), and a control group that was not jetlagged but was fed a night-time restricted feeding diet (Ctrl RF). Mice that received TRF had ad libitum access to normal chow for 12 consecutive hours during the dark (=active) period of the 12:12 LD cycle of control mice. After 12 hours, the chow was removed for the next 12 hours during the light (=inactive) period of the 12:12 LD cycle of the control mice to mimic the food intake pattern of control mice fed ad libitum that consume most of their food intake during the dark phase. Body mass was monitored at the time of sacrifice and once a week at the second day of the normal light/dark cycle (ZT 0 = lights on) in order to avoid any acute effect of the time-shift during the four weeks of jetlag induction. Food intake was measured in the fourth week of jetlag induction at the second day of the normal light/dark cycle. After four weeks of jetlag induction, the mice were sacrificed over the course of 24 h at 4 h intervals. Jetlagged mice were sacrificed when the jetlagged mice were in the same light/dark cycle as control mice, and ZTs were synchronized (i.e., ZT 0 of jetlag mice corresponded to ZT 0 of control mice). The euthanization of the mice from the different groups and timepoints was randomized over the second, third, and fourth days of the period that the control and jetlagged mice were in the same light/dark cycle. The luminal content of the distal colon was collected for measurement of SCFA concentrations and stored at −80 °C. Blood was collected via cardiac puncture and processed for plasma ghrelin measurements. Blood glucose was measured in cardiac blood using a glucometer. Distal colonic mucosa and hypothalamic tissue were isolated, stored in RNAlater (Qiagen, Hilden, Germany), and processed for quantitative real-time PCR (qRT-PCR). All mice (N = 8) were included at each timepoint in the final analyses unless stated otherwise.

### 2.3. Analysis of Fecal SCFA Concentrations

Fecal samples (100 mg) were suspended in 1 mL of saturated NaCl (36%) solution. An internal standard (50 μL 2-ethylbutyric acid) was added, and the samples were homogenized using glass beads. SCFAs were extracted with ether (3 mL) in the presence of H_2_SO_4_ (150 μL). The ether layer was collected and dried by Na_2_SO_4_ (50 mg). Analysis was performed by gas chromatography–flame ionization detector (Agilent, Santa Clara, CA, USA), with an injection volume of 0.5 μL. The resulting chromatograms were processed using the Xcalibur software (Thermo Fischer Scientific, Waltham, MA, USA).

### 2.4. Quantitative Real-Time PCR

Total RNA from the distal colonic mucosa and hypothalamus was isolated using the RNeasy Mini Kit (Qiagen, Hilden, Germany). The isolated total RNA was treated with the Turbo DNA-free^TM^ kit (Thermo Fisher Scientific, Waltham, MA, USA) and reverse transcribed to cDNA using qScript cDNA SuperMix (Quanta BioSciences, Gaithersburg, MD, USA) according to the manufacturer’s instructions. qRT-PCR was performed using the Lightcycler 480 (Roche Diagnostics, Basel, Switzerland) with the Lightcycler 480 Sybr Green I Master mix (Roche Diagnostics, Basel, Switzerland). A calibrator was used to correct for inter-run variability between plates. Results were expressed relative to the geometric mean of the normalized expression of three stable housekeeping genes, determined according to the method of Vandesompele, which did not show a circadian rhythm [39] (distal colonic mucosa: *β-actin*, TATA box binding protein (*Tbp*), and cyclophilin (*Cycloph*); hypothalamus: *Cycloph, Tbp*, and hydroxymethylbilane synthase (*Hmbs*)). Primer sequences are shown in Table 1.

### 2.5. Plasma Hormone Measurements

Plasma samples for total and octanoylated ghrelin were acidified (0.1 N HCl) and supplemented with AEBSF to a final concentration of 80 mmol/L (Sigma-Aldrich, Saint Louis, MO, USA), extracted on a Sep-Pak C18 column (Waters Corporation, Milford, MA, USA), and vacuum-dried. The radioimmunoassay for total and octanoyl ghrelin was performed using an in-house-developed radioimmunoassay as previously described [40]. Plasma corticosterone levels were detected using the Corticosterone EIA Kit (K014-H1; Arbor Assays^®^, Ann Arbor, MI, USA) according to the manufacturer’s protocol. 

### 2.6. Statistical Analysis

Results are presented as mean ± SEM, unless stated otherwise. All statistical analyses were performed in SAS Studio University Edition 9.4. Comparison of body mass between Ctrl RF, JL AL, and JL RF mice over time was performed using a linear mixed model with time as random factor, mouse as subject, and group and time as fixed factor, followed by planned comparison and post hoc testing in Proc Mixed. Comparison of food intake between Ctrl RF, JL AL, and JL RF mice over time was performed using a linear mixed model with time and day/night as random factors; cage as subject; and group, time, and day/night as fixed factors, followed by planned comparison and post hoc testing in Proc Mixed. Since the qPCR data were distributed in a non-normal and/or non-homogeneous manner, we used log-transformed data for all further analyses of the qPCR data. Diurnal rhythm analysis in the jetlag model was calculated using the cosinor procedure developed in SAS, in which the best-fitting cosine curve for a data set was calculated using a non-linear regression model in proc NLIN [41]. Probability values for the best fitting cosine curve are indicated as *p*_cosinor_. Differences in acrophase (the time point where the fitted cosine curve reaches its maximum); mesor; and amplitude between Ctrl RF, JL AL, and JL RF mice within one output measure were compared using a non-linear regression model in Proc NLMixed. Differences in acrophase between 2 different output measures was performed using a non-linear mixed regression model with mouse as subject in Proc NLMixed. Significance was accepted at the 5% level.

## 3. Results

### 3.1. TRF Selectively Restored the Disruption of the Central Circadian Clock during Chronic Jetlag

**Figure 2:**
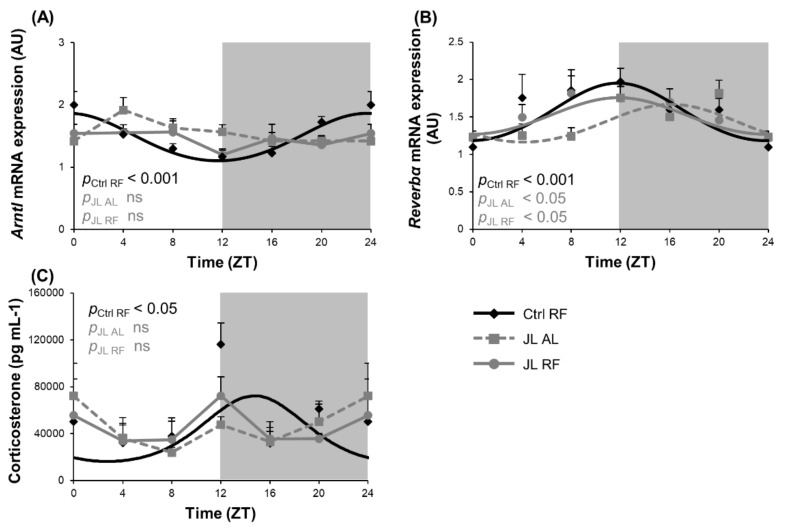
Effect of time restricted feeding on hypothalamic clock gene mRNA expression and plasma corticosterone levels. (**A**) Arntl and (**B**) Reverbα mRNA expression in the mouse hypothalamus (*n* = 8 mice per time point per group) of control mice fed a TRF diet (Ctrl RF), jetlagged mice fed ad libitum (JL AL) and jetlagged mice fed a TRF diet (JL RF). (**C**) Plasma corticosterone levels (*n* = 4 mice per time point per group). The fitted cosine curve determined by cosinor analysis (period = 24 h) are shown in Ctrl RF, JL AL and JL RF mice. The dark phase is shaded grey. Statistics: Non-linear regression (Proc NLIN). ns = not significant. p values refer to probability values for the best fitting cosine curve.

Altering the light/dark cycle is known to impair the central circadian clock. We investigated whether TRF could avert the effect of disruption of the light/dark cycle during chronic jetlag on the rhythmic expression of clock genes in the hypothalamus. *Arntl* mRNA expression showed diurnal rhythmicity (*p*_cosinor_ < 0.001) in the hypothalamus of the Ctrl RF mice. The acrophase or zeitgeber time (ZT) at which the rhythm peaked was ZT 23h38. This rhythm was abolished in both the JL AL group and the JL RF group (Figure 2A). In contrast, *Reverbα* mRNA expression displayed a diurnal rhythm in the Ctrl RF group (*p*_cosinor_ < 0.001), peaking at ZT 11h47, which was phase delayed by 4h20 in the JL AL group (ZT 16h07; *p*_cosinor_ < 0.05) and restored by TRF in the JL RF mice (JL RF: ZT 11h50, *p*_cosinor_ < 0.05) (Figure 2B). Plasma corticosterone levels, which ensure communication between the central and peripheral clocks and act as a read out for the condition of the master clock, were measured as well. Plasma corticosterone levels were diurnal (ZT 14h46; *p*_cosinor_ < 0.05) in the Ctrl RF group but were abolished in the JL AL. Similar to the effects on *Arntl* mRNA expression, TRF could not restore the loss in rhythmicity induced by chronic jetlag (Figure 2C). Table 2 compares the changes in the acrophase, amplitude, and mesor (=average over 24 h) of all the measured parameters of the study in the three groups.

### 3.2. TRF Prevented the Increase in Body Mass Induced by Chronic Jetlag

**Figure 3:**
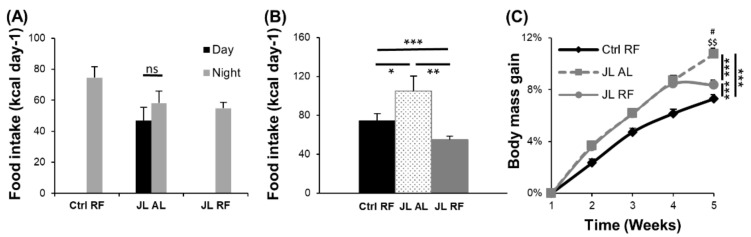
Effect of time-restricted feeding on food intake and body mass. (**A**) Day/night food intake pattern of control mice fed a TRF diet (Ctrl RF, N = 48), jetlagged mice fed ad libitum (JL AL, N = 48), and jetlagged mice fed a TRF diet (JL RF, N = 48) at week 4. (**B**) Total 24 h caloric intake in Ctrl RF (N = 48), JL AL (N = 48), and JL RF (N = 48) mice at week 4. (**C**) Percentage increase in body mass in Ctrl RF (N = 48), JL AL (N = 48), and JL RF (N = 48) mice. Data are presented as mean ± SEM. Statistics: mixed model analysis followed by planned comparison and post hoc testing. The interaction effects between the different groups over 5 weeks are depicted as * *p* < 0.05; ** *p* < 0.01; *** *p* < 0.001. The difference in body mass between the groups at week 5 are depicted as # *p* < 0.05 compared to JL AL; $$ *p* < 0.01 compared to Ctrl RF.

Disruption of the central clock and consequently of peripheral clocks may affect the food intake pattern during chronic jetlag. Indeed, while mice fed ad libitum normally consume most of their calories during the active phase (70%), caloric intake was similar during day and night in JL AL mice (Figure 3A). Total daily food intake was significantly different between all three groups. JL AL mice consumed more calories on a daily basis compared to Ctrl RF mice (*p* < 0.05) and the JL RF mice (*p* < 0.01), while JL RF mice consumed less calories than Ctrl RF mice (*p* < 0.001) (Figure 3B). These differences in caloric intake affected body mass between the three groups (time × group: *p* < 0.001). JL AL mice gained more weight than the Ctrl RF mice, but in the JL RF mice, this was prevented after 4 weeks of TRF. Indeed, at week 5, the increase in body mass in the JL RF group was significantly (*p* < 0.05) less than in the JL AL group and did not differ any more from the Ctrl RF group (Figure 3C).

### 3.3. TRF Partially Prevented the Changes in Rhythmicity of Plasma Ghrelin and Blood Glucose Levels Caused by Chronic Jetlag

**Figure 4:**
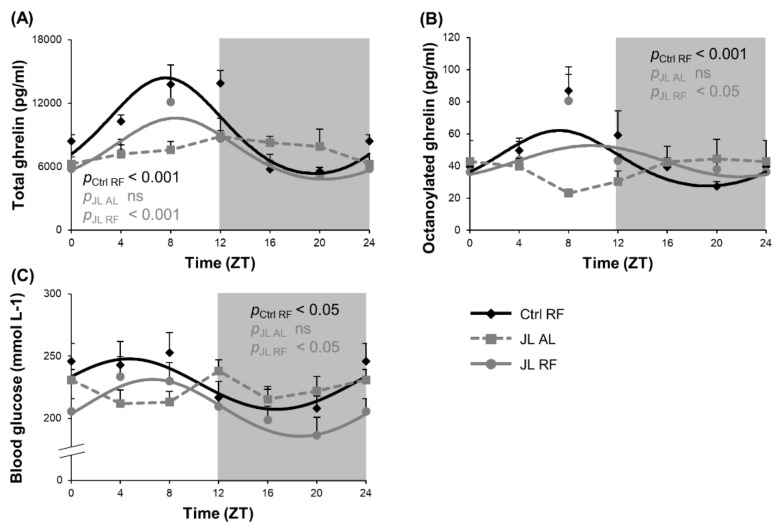
Effect of time restricted feeding on diurnal fluctuations in plasma ghrelin levels and blood glucose levels. Plasma (**A**) total ghrelin, (**B**) octanoylated ghrelin and (**C**) blood glucose levels in control mice fed a TRF diet (Ctrl RF), jetlagged mice fed ad libitum (JL AL) and jetlagged mice fed a TRF diet (JL RF) (*n* = 8 mice per time point per group). The fitted cosine curve determined by cosinor analysis (period = 24 h) are shown. The dark phase is shaded grey. Statistics: Non-linear regression (Proc NLIN). ns = not significant. *p* values refer to probability values for the best fitting cosine curve.

We postulate that TRF may uncouple central and peripheral clocks and entrain ghrelin as a food entrainable oscillator to entrain food anticipatory behavior and food intake during TRF. Both plasma total and octanoylated (=active) ghrelin levels showed a diurnal rhythm in the Ctrl RF group, which peaked at ZT 8h03 (*p*_cosinor_ < 0.001) and ZT 7h36 (*p*_cosinor_ < 0.001), respectively. Both diurnal ghrelin rhythms were lost in the JL AL group and could be avoided by TRF in the JL RF mice. The acrophases of plasma ghrelin levels of the JL RF mice (total: ZT 8h04, *p*_cosinor_ < 0.001; octanoylated: ZT 9h49, *p*_cosinor_ < 0.05) did not differ significantly from the Ctrl RF group. However, the rhythm in plasma total ghrelin levels in the JL RF was dampened with 32% (*p* < 0.05), and the average plasma total ghrelin level over 24 h was decreased (*p* < 0.001) compared to the Ctrl RF mice. In contrast, the plasma octanoylated ghrelin levels were not dampened by TRF and did not differ overall over 24 h (Figure 4A,B). Further, the acrophase of the plasma active ghrelin levels and hypothalamic *Reverbα* mRNA expression (ZT 11h50) did not differ significantly (non-linear mixed model with mesor as random effect) in JL RF mice, suggesting that ghrelin might represent one of the feedback signals from the periphery to the central circadian clock.

Blood glucose levels were measured as an additional metabolic parameter to determine the effect of preventing the disruption of the rhythm in food intake induced by chronic jetlag using TRF. A diurnal rhythm in blood glucose levels was observed in the Ctrl RF group (*p*_cosinor_ < 0.05), peaking at ZT 4h42, which was abolished in the JL AL group (Figure 4C). In the JL RF group, a diurnal rhythm (*p*_cosinor_ < 0.05) was observed that peaked in phase ZT 6h37 with the control group. However, JL RF mice (207.22 ± 4.45 mmol L^−1^) had lower average blood glucose concentrations over 24 h compared to the Ctrl RF mice (226.65 ± 4.92 mmol L^−1^) (*p* < 0.01), probably resulting from the decreased total daily food intake in JL RF mice.

### 3.4. TRF Prevented the Rhythmicity in Npy mRNA Expression in the Hypothalamus That Was Abolished by Chronic Jetlag

**Figure 5:**
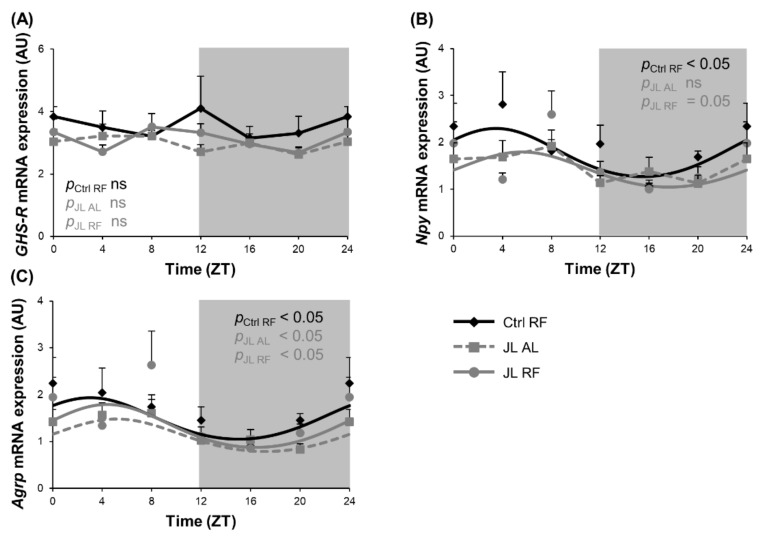
Time restricted feeding prevents the rhythmicity in hypothalamic Npy mRNA expression that is abolished by chronic jetlag. (**A**) GHS-R, (**B**) Npy and (**C**) Agrp mRNA expression in the mouse hypothalamus of control mice fed a TRF diet (Ctrl RF), jetlagged mice fed ad libitum (JL AL) and jetlagged mice fed a TRF diet (JL RF) (*n* = 8 mice per time point per group). The fitted cosine curve determined by cosinor analysis (period = 24 h) are shown in Ctrl RF, JL AL and JL RF mice. The dark phase is shaded grey. Statistics: Non-linear regression (Proc NLIN). ns = not significant. *p* values refer to probability values for the best fitting cosine curve.

Ghrelin induces food intake by stimulating the release of the neuropeptides NPY and AgRP via activation of the ghrelin receptor growth hormone secretagogue receptor (GHS-R) in the hypothalamic arcuate nucleus. We hypothesized that prevention of the loss in rhythmicity in plasma ghrelin levels during TRF in jetlagged mice would also prevent alterations in diurnal fluctuations in *Npy*/*Agrp* mRNA expression induced by chronic jetlag. *GHS-R* mRNA expression was not rhythmic in all three groups, with no differences between the groups (Figure 5A). Hypothalamic *Npy* mRNA expression showed a diurnal rhythm in the Ctrl RF group (ZT 3h29, *p*_cosinor_ < 0.05) that was abolished in the JL AL and prevented by TRF in the JL RF mice (JL RF: ZT 5h37, *p*_cosinor_ = 0.05) (Figure 5B). However, the acrophase of the plasma ghrelin levels and hypothalamic *Npy* mRNA expression in both Ctrl RF and JL RF mice differed significantly (non-linear mixed model with mesor as random effect; *p* < 0.01). Overall *Npy* mRNA expression over 24 h was decreased (*p* < 0.05) by 20% in the JL RF mice compared to the Ctrl RF mice, which is in agreement with the decreased caloric intake in the JL RF (Figure 3B). *Agrp* mRNA expression showed diurnal rhythmicity in all three groups (*p*_cosinor_ < 0.05), peaking around ZT 4. The rhythms were not significantly different from each other (Figure 5C). However, chronic jetlag decreased the overall *Agrp* mRNA expression over 24 h compared to the Ctrl RF (*p* < 0.05) mice that was prevented by TRF in the JL RF mice. 

### 3.5. TRF Prevented Alterations in Diurnal Fluctuations in Fecal SCFA Concentrations Caused by Chronic Jetlag

**Figure 6:**
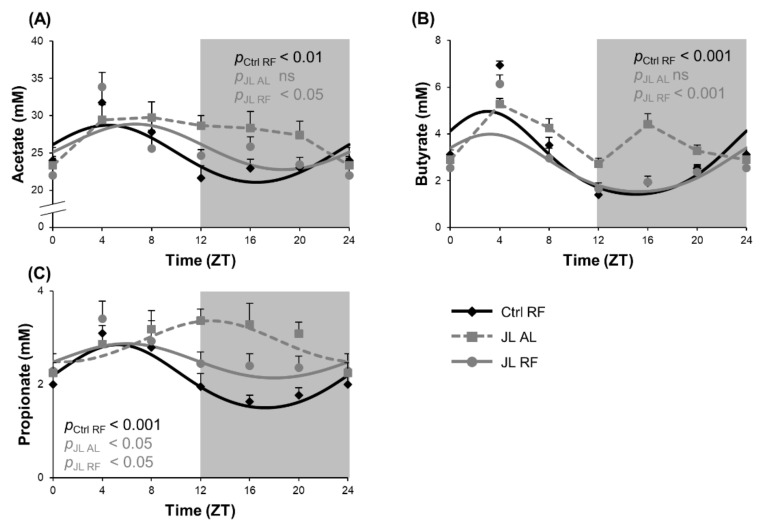
Effect of time restricted feeding on diurnal fluctuations in fecal SCFA concentrations. Fecal (**A**) acetate, (**B**) butyrate and (**C**) propionate concentrations in the distal colon of control mice fed a TRF diet (Ctrl RF), jetlagged mice fed ad libitum (JL AL) and jetlagged mice fed a TRF diet (JL RF) (*n* = 8 mice per time point per group). The fitted cosine curve determined by cosinor analysis (period = 24 h) are shown. The dark phase is shaded grey. Statistics: Non-linear regression (Proc NLIN). ns = not significant. *p*-values refer to probability values for the best fitting cosine curve.

We hypothesized that imposing a normal rhythmic food intake pattern in jetlagged mice could prevent changes in diurnal fluctuations in SCFA levels in the colon induced by chronic jetlag. Fecal concentrations of acetate and butyrate showed a diurnal rhythm in the Ctrl RF group (acetate: ZT 4h28, *p*_cosinor_ < 0.01; butyrate: ZT 3, *p*_cosinor_ < 0.001) that was lost in the JL AL group and prevented by TRF in the JL RF group (acetate: ZT 6h39, *p*_cosinor_ < 0.05; butyrate ZT 3h13, *p*_cosinor_ < 0.001). The latter did not differ significantly from the Ctrl RF group (Figure 6A,B). Further, fecal propionate concentrations also showed a diurnal rhythm in the Ctrl RF group (*p*_cosinor_ < 0.001), peaking at ZT 5h44. In contrast to fecal acetate and butyrate concentrations, in the JL AL group, the fecal propionate concentrations still showed a diurnal rhythm (*p* < 0.05) but the rhythm was phase delayed by 7h04 compared to the Crtl RF group (*p* < 0.001). In the JL RF group, the shift in rhythm was prevented (ZT 5h35, *p*_cosinor_ < 0.05) and did not differ significantly from the Ctrl RF group (Figure 6C). In addition, the average fecal propionate concentration was increased by 36% in the JL AL mice (*p* < 0.001) and by 22% in the JL RF mice over 24 h (*p* < 0.001) compared to the Ctrl RF mice. No difference was observed in average fecal acetate and butyrate concentrations over 24 h between all three groups.

### 3.6. TRF Partially Prevented Changes in Clock Gene Expression of Colonic Mucosa Caused by Chronic Jetlag

**Figure 7:**
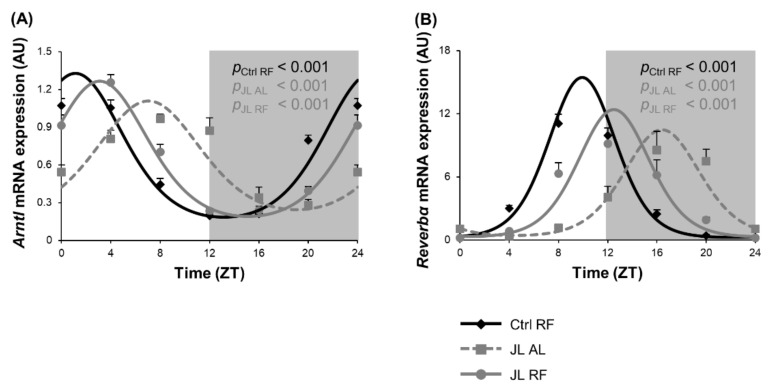
Effect of time restricted feeding on clock gene mRNA expression in the colonic mucosa. (**A**) Arntl and (**B**) Reverbα mRNA expression in the mouse distal colonic mucosa of control mice fed a TRF diet (Ctrl RF), jetlagged mice fed ad libitum (JL AL) and jetlagged mice fed a TRF diet (JL RF) (N = 8 mice per time point per group). The fitted cosine curve determined by cosinor analysis (period = 24 h) are shown in Ctrl RF, JL AL and JL RF mice. The dark phase is shaded grey. Statistics: Non-linear regression (Proc NLIN). *p*-values refer to probability values for the best fitting cosine curve.

We previously showed that microbial metabolites such as SCFAs are known to entrain circadian clock gene expression in the colonic mucosa [16,42]. Therefore, circadian clock gene expression was studied in the mucosa of the distal colon over 24 h in Ctrl RF, JL AL, and JL RF mice. *Arntl* mRNA expression showed diurnal rhythmicity (*p*_cosinor_ < 0.001) in all three groups. The rhythm in *Arntl* mRNA expression in the JL AL group (ZT 7h02) was phase delayed by 5h55 (*p* < 0.001), and the amplitude was dampened (*p* < 0.01) compared to the Ctrl RF group (ZT 1h07). TRF could partially prevent the phase delay but did not normalize the rhythm in *Arntl* mRNA expression in the JL RF group (ZT 3h05, *p*_cosinor_ < 0.001). However, the dampening was completely prevented by TRF in the JL RF mice (Figure 7A). *Reverbα* mRNA expression was also diurnal in all three groups (*p*_cosinor_ < 0.001), peaking at ZT 9h54 in the Ctrl RF mice, ZT 16h23 in the JL AL mice, and ZT 12h29 in the JL RF mice. Chronodisruption induced, similar to *Arntl*, a phase delay of 6h31 (*p* < 0.001) and a dampening of the amplitude (*p* < 0.05) compared to the Ctrl RF group. TRF in the jetlagged mice could prevent the dampening but only partially prevented the shift in *Reverbα* mRNA expression (Figure 7B).

## 4. Discussion

In the present study, we showed that chronic jetlag impaired the central circadian clock, affecting the plasma corticosterone levels that convey the circadian information from the light/dark cycle to the peripheral circadian clocks. TRF selectively prevents the disruption of the central circadian clock as hypothalamic clock gene expression of *Reverbα*, but not of *Arntl* and corticosterone secretions were restored. During TRF in chronic jetlagged mice, food cues become the dominant zeitgeber preventing the loss in rhythmicity in plasma ghrelin levels and in hypothalamic *Npy* mRNA expression in order to promote food intake. Avoiding the disruption of the food intake pattern induced by chronodisruption counters the increase in body mass observed during chronodisruption. TRF during chronodisruption prevented the changes in the rhythmicity of fecal SCFAs levels, thereby resynchronizing the rhythmic activation of the peripheral circadian clocks. 

To evaluate whether food as an entraining factor in the gut could feedback to the hypothalamus to restore the central circadian clock and its output signals, like corticosterone, we measured circadian clock genes in the hypothalamus and plasma corticosterone levels. TRF could not counteract the loss in the diurnal rhythm of *Arntl* mRNA expression in the hypothalamus but prevented the phase delay in the rhythm of *Reverbα* mRNA expression induced by chronic jetlag in the JL RF mice. Strong heterogeneity of entrainment kinetics has already been shown not only between different organs, but also within the molecular clockwork of each tissue [43,44]. Thus, it is possible that if the study lasted longer, *Arntl* expression could also have been restored in the hypothalamus. Corticosterone secretion is known to be tightly controlled by the SCN and is therefore a peripheral parameter to evaluate the condition of the central circadian clock [45,46]. TRF in the JL RF mice could not prevent the loss in diurnal rhythmicity induced by jetlag, which is in agreement with the fact that the rhythm of the core clock gene, *Arntl*, was not restored. 

Several studies show that chronodisruption (genetic or environmental) disrupts the rhythms in food intake in mice [14,16,32]. This is in agreement with our results showing that the JL AL mice consumed equal amounts of calories during the day and night. In addition, the daily consumed calorie intake differed between all three groups. JL RF mice consumed less calories than Ctrl RF mice, probably because they failed to adapt to the new feeding schedule because of the jetlag induction every 3 to 4 days. Ren et al. [47] already showed that mice that were fed a TRF diet (only access to food in the dark phase) and exposed to a single (only one day) six hours advanced jetlag needed on average 3–7 days to adapt to the new feeding schedule. 

The increased daily caloric intake of JL AL mice compared to Ctrl RF mice increased body mass and could be prevented using TRF during chronodisruption. These results are consistent with studies showing that chronodisrupted high-fat diet-induced obese mice, subjected to a TRF schedule, in both a preventive and therapeutic manner, did not gain significantly more weight than Ctrl mice that were fed ad libitum [23,26]. In whole-body *Cry1* and *Cry2*, as well as in liver-specific *Arntl* and *Reverbα/β* knockout mice, TRF protected mice from excessive weight gain and metabolic diseases compared to both WT and clock gene KO mice that were fed ad libitum. In the same study, TRF only led to a significant lower body mass compared to the ad libitum fed mice, starting 7 weeks after commencing the feeding regimen [5]. This is in agreement with our results as a difference between the JL AL and JL RF mice became only apparent at week 5. In humans, studies showed that intermittent fasting (IF), that is, a form of time-restricted eating (TRE), has several positive effects on obesity (reducing body mass/fat) and obesity-related diseases such as lowering of blood pressure, improvement of insulin sensitivity with a reduction of glucose and/or insulin levels, and improvement of lipid profiles [25,48,49,50]. 

Studies in SCN-lesioned mice showed that the central circadian clock regulates the feeding/fasting cycle [51,52]. During chronodisruption, food, using TRF, becomes the major zeitgeber and further uncouples the central and peripheral clocks. Our findings show that TRF during chronic jetlag counters the loss in diurnal fluctuations in plasma ghrelin levels that act as a food entrainable oscillator to resynchronize food anticipatory behavior and food intake. LeSauter et al. [53] previously showed that injection of ghrelin increased food anticipatory activity and food intake, and suggested that ghrelin is the food entrainable oscillator that stimulates food intake. The peak in plasma ghrelin levels (ZT 9h49) before the start of the TRF feeding (ZT12) in JL RF mice confirms these findings. Similarly, chronodisruption induced by a HFD was also previously shown to abolish the food anticipatory increase in plasma ghrelin levels [10]. 

The acrophase of the plasma ghrelin levels (ZT 9h49) and hypothalamic *Reverbα* mRNA expression (ZT 11h50) did not differ significantly in JL RF mice. It is therefore tempting to speculate that plasma ghrelin feeds back to the central circadian clock to partially restore the rhythmicity in hypothalamic *Reverbα* but not *Arntl* mRNA expression during TRF. Indeed, ghrelin has been shown to phase advance spontaneous firing in SCN slices and the rhythm of PER2::LUC expression in cultured SCN explants expressing the ghrelin receptor [54,55,56]. Injection of the ghrelin mimetic, GHRP-6, 30 h after food deprivation induced a phase advance in locomotor activity [54].

Ghrelin exerts its orexigenic function by binding to the ghrelin receptor in the arcuate nucleus and stimulating the release of NPY and AgRP [57,58,59,60]. TRF during chronic jetlag prevented the loss in the rhythmicity in hypothalamic *Npy* expression peaking at ZT 5h37 in JL RF mice similar as in the Ctrl RF mice (ZT 3h29). This acrophase was significantly different from ghrelin (ZT 9h49), indicating that the peak in *Npy* expression is not directly triggered by ghrelin but occurs during the light phase to replenish the depleted NPY storage after being secreted during the period of food intake in the dark phase. A limitation of our study is that we did not measure changes in NPY protein expression that might fluctuate in parallel with ghrelin. A study in a *Arntl* KO hypothalamic cell line showed that ARNTL binds to the *Npy* promotor region, but not of *Agrp* or *Pomc*, upon stimulation with bisphenol A or palmitate [61,62]. This could possibly explain the loss in rhythm in hypothalamic *Npy*, but not *Agrp* mRNA expression, which paralleled the loss in hypothalamic *Arntl* expression during chronic jetlag. Nonetheless, TRF during chronic jetlag could not restore rhythmicity in hypothalamic *Arntl* expression, while it did prevent the loss in rhythmicity in *Npy* expression. It could be that in the absence of a properly function circadian clock food cues become an entraining factor for *Npy* expression. However, Cedernaes et al. [63] showed that mice with an AgRP-specific ablation of *Arntl* exhibited a significant reduction in food intake during the dark period and an increased intake during the light period. This is not consistent with our results, as *Agrp* mRNA expression is not affected in the JL AL mice despite a loss in the rhythmicity in *Arntl* expression.

Preventing the disruption of the food intake pattern in jetlagged mice was also reflected in the fluctuations in blood glucose levels. It was previously shown that a disturbed diurnal eating pattern induced by a disrupted circadian clock resulted in alterations in glucose metabolism in rats [64]. In overweight adults, eTRF (eating between 8 h and 14 h) also improved 24 h glucose levels [22]. 

SCFAs show diurnal fluctuations that are affected by alterations in the food intake pattern/diet such as TRF [16,36,65]. TRF during chronic jetlag prevented the loss/shift in rhythmicity of fecal SCFA levels. Segers et al. [66] already showed that in *Arntl*^−/−^ mice, the rhythm of SCFA was absent and could be avoided using TRF. The loss in rhythmicity in SCFA levels in the *Arntl*^−/−^ mice was probably due to the loss of rhythmicity in the food intake pattern as well [32]. Studies in both human and mice showed that TRF intervention alters both microbial abundance, composition, and metabolites to alleviate several diseases [67,68,69,70]. Taken together, our findings suggest that the changes in the rhythmicity in fecal SCFA levels during chronic jetlag are indeed a result of the disrupted food intake pattern and can be prevented using TRF.

We previously showed that SCFAs can entrain the transcript expression of *Arntl*, *Reverbα*, and *Per2* in primary colonic crypt cultures [16]. Oral administration of SCFAs also has been shown to shift the circadian clock in the liver and kidney in mice [42]. Our current findings show that TRF during chronic jetlag partially prevented the phase delay and countered the decrease in the amplitude of clock genes in the gut mucosa. Some studies already illustrated the effect of TRF on peripheral clock genes. For example, Jamshed et al. [22] showed that eTRF in humans (a six hour eating window between 8 and 14 h) increased the expression of several clock genes such as *Arntl*, *Per1*, *Cry1*, *Cry2*, *Reverbα*, and *RORα* in the blood at 20 h compared to 8 h [71,72]. Taken together, these findings suggest that the food intake pattern, most likely by affecting the microbial production of SCFA, is indeed an entraining signal for clock genes in the colon. It is possible that if the study lasted longer, the changes in peripheral clock gene expression that are caused by chronic jetlag could be completely prevented. Further, other microbial metabolites such as secondary bile acids have also been shown both in vitro and in vivo to entrain the peripheral circadian clocks [38]. Besides microbial metabolites, metabolism can also affect the circadian clock. Hormones such as insulin, leptin, ghrelin, and glucagon have been demonstrated to acutely affect circadian clock expression [3,73,74,75]. Thus, it is possible that countering the change in rhythmicity in the SCFA levels alone is not sufficient to prevent the shifts in the circadian clock, but also other metabolic or microbial cues are necessary.

Our results suggest that maintaining a normal food intake pattern during chronodisruption prevents the disruption of the peripheral circadian clocks and the concomitant increase in body mass. Thus, shift-workers can prevent or ameliorate health issues linked to chronodisruption by restricting calorie intake to the light (normal active) phase of the day. Possibly pre- and/or probiotics can be used to further restore the normal peak in fecal SCFAs, although this should be studied in more detail.

In conclusion, our findings suggest that food is a strong entraining cue during chronodisruption that can prevent loss in the rhythmicity in plasma ghrelin levels and hypothalamic *Npy* expression that regulate the day/night food intake rhythm. As a result, fecal SCFA levels will continue to fluctuate thereby resynchronizing the peripheral circadian clock in the gut. Further, food-induced cues might indirectly feedback to the central circadian clock to restore clock gene expression in the hypothalamus. Thus, TRF could be a good non-invasive measure to prevent certain aspects of chronodisruption that are caused by chronic jetlag.

## Figures and Tables

**Table 1:** Primers used for qRT-PCR.

Gene	Forward Primer Sequence	Reverse Primer Sequence
*β-actin*	CCTGTGCTGCTCACCGAGGC	GACCCCGTCTCTCCGGAGTCCATC
*Cycloph*	GGAGATGGCACAGGAGGAAA	CCCGTAGTGCTTCAGCTTGAA
*Hmbs*	CTGAAGGATGTGCCTACCATAC	AAGGTTTCCAGGGTCTTTCC
*Tbp*	AGGATGCTCTAGGGAAGAT	TGAATAGGCTGTGGAGTAAGT
*Npy*	CCGCTCTGCGACACTACAT	TGTCTCAGGGCTGGATCTCT
*Agrp*	GCGGAGGTGCTAGATCCA	AGGACTCGTGCAGCCTTA
*GHS-R*	TCAGGGACCAGAACCACAAA	CCAGCAGAGGATGAAAGCAA
*Arntl*	CGTTTCTCGACACGCAATAGAT	TCCTGTGGTAGATACGCCAAAA
*Reverbα*	CCCTGGACTCCAATAACAACACA	GCCATTGGAGCTGTCACTGTAG

**Table 2:** Cosinor values of all measured parameters.

			Condition	*p* Value Cosinor	Acrophase (h) (±SD)	Amplitude	Mesor (±SD)
Hypothalamus	Circadian Clock	*Arntl*	Ctrl RF	<0.001	23h38 (±0h46)	0.38	1.44 (±0.05)
			JL AL	ns	NA	NA	NA
			JL RF	ns	NA	NA	NA
		*Reverbα*	Ctrl RF	<0.001	11h47 (±0h56)	0.39	1.52 (±0.07)
			JL AL	<0.05	16h07 (±1h11) **	0.25	1.39 (±0.06)
			JL RF	<0.05	11h50 (±1h23) #	0.25	1.49 (±0.06)
	*GHS-R*		Ctrl RF	NA	NA	NA	NA
			JL AL	NA	NA	NA	NA
			JL RF	NA	NA	NA	NA
	*Npy*		Ctrl RF	<0.05	3h29 (±1h19)	0.52	1.71 (±0.13)
			JL AL	ns	NA	NA	NA
			JL RF	=0.05	5h37 (±1h22)	0.38	1.37 (±0.10) *
	*Agrp*		Ctrl RF	<0.05	3h02 (±1h24)	0.44	1.43 (±0.12)
			JL AL	<0.05	5h10 (±1h16)	0.35	1.08 (±0.08) *
			JL RF	<0.05	4h24 (±1h07)	0.46	1.25 (±0.10)
Plasma	Corticosterone		Ctrl RF	<0.05	14h46 (±1h16)	27996.33	34348.46 (±5488.43)
			JL AL	ns	NA	NA	NA
			JL RF	ns	NA	NA	NA
	Total ghrelin		Ctrl RF	<0.001	8h03 (±0h29)	4073.69	9306.82 (±351.71)
			JL AL	ns	NA	NA	NA
			JL RF	<0.001	8h04 (±0h37)	2765.73 *	7359.93 (±312.97) ***
	Octanoylated ghrelin		Ctrl RF	<0.001	7h36 (±0h46)	16.99	43.89 (±2.42)
			JL AL	ns	NA	NA	NA
			JL RF	<0.05	9h49 (±1h16)	9.73	43.07 (±2.27)
	Glucose		Ctrl RF	<0.05	4h42 (±1h18)	18.88	226.65 (±4.92)
			JL AL	ns	NA	NA	NA
			JL RF	<0.05	6h37 (±1h03)	22.88	207.22 (±4.45) **
Fecal SCFA	Acetate		Ctrl RF	<0.01	4h28 (±0h57)	3.84	24.62 (±0.67)
			JL AL	ns	NA	NA	NA
			JL RF	<0.05	6h39 (±1h14)	3.08	25.64 (±0.71)
	Butyrate		Ctrl RF	<0.001	3h00 (±0h32)	1.78	2.66 (±0.16)
			JL AL	ns	NA	NA	NA
			JL RF	<0.001	3h13 (±0h41)	1.23	2.47 (±0.15)
	Propionate		Ctrl RF	<0.001	5h44 (±0h41)	0.68	2.11 (±0.86)
			JL AL	<0.05	12h48 (±1h24) ***	0.44	2.89 (±0.12) ***
			JL RF	<0.05	5h35 (±1h12) ###	0.49	2.58 (±0.11) ***
	Total SCFA		Ctrl RF	<0.001	4h14 (±0h45)	2.92	29.68 (±0.88)
			JL AL	ns	NA	NA	NA
			JL RF	<0.05	5h51 (±1h04)	4.70	30.99 (±0.93)
Distal Colonic Mucosa	Circadian Clock	*Arntl*	Ctrl RF	<0.001	1h07 (±0h11)	0.57	0.50 (±0.02)
			JL AL	<0.001	7h02 (±0h15) ***	0.44 **	0.52 (±0.02)
			JL RF	<0.001	3h05 (±0h12) *** ###	0.54 #	0.49 (±0.02)
		*Reverbα*	Ctrl RF	<0.001	9h54 (±0h11)	7.60	1.97 (±0.14)
			JL AL	<0.001	16h23 (±0h14) ***	5.03 *	2.10 (±0.15)
			JL RF	<0.001	12h29 (±0h12) *** ###	6.03	2.01 (±0.15)

ns = not significant, NA = not applicable/no rhythm, Ctrl RF = control mice fed a TRF diet, JL AL = jetlagged mice fed ad libitum, JL RF = jetlagged mice fed a TRF diet. Statistics: non-linear regression (Proc NLMixed). * *p* < 0.05 compared to Ctrl RF, ** *p* < 0.01 compared to Ctrl RF, *** *p* < 0.001 compared to Ctrl RF, # *p* < 0.05 compared to JL AL, ### *p* < 0.001 compared to JL AL.

## Data Availability

The data that support the findings of this study are available from the corresponding author upon reasonable request.
